# Supplementary material for: Pantothenate Kinase 4 Governs Lens Epithelial Fibrosis by Negatively Regulating Pyruvate Kinase M2-Related Glycolysis
Source: Aging Dis. 2023 Oct 1;14(5):1834–52. doi: 10.14336/AD.2023.0216-1 (PMC10529755; doi:10.14336/AD.2023.0216-1)
Supplement: Supplementary file 1 [file AD-14-5-1834-s.pdf]

SUPPLEMENTARY DATA

Pantothenate Kinase 4 Governs Lens Epithelial Fibrosis by Negatively Regulating Pyruvate Kinase M2-Related Glycolysis

Xue Li , Lin-Lin Luo, Rui-Feng Li , Chun-Lin Chen, Min Sun*, Sen Lin*

© 2022. Li X et al. Published online at http://www.aginganddisease.org/EN/10.14336/AD.2023.0216-1

SUPPLEMENTARY DATA

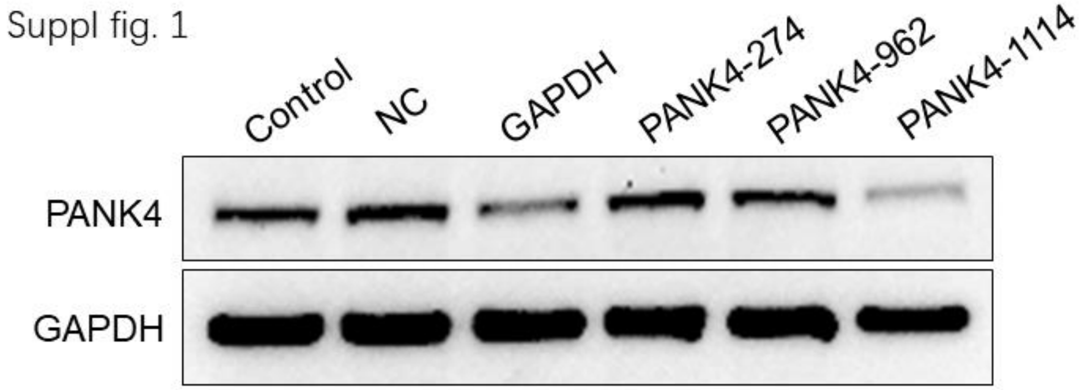

Figure 1. **Screen assay of 3 types of designed siPank4 by Western blot.**

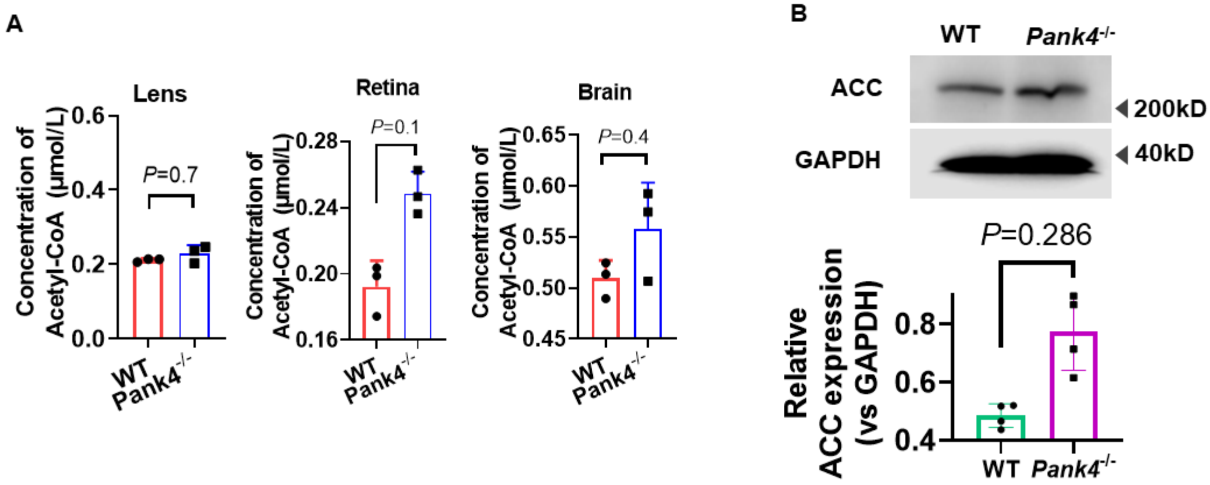

Figure 2. (**A**) Concentrations of acetyl-CoA in WT and *Pank4*$^{-/-}$ mice lens epithelium, brain cortex and retina. Data were analyzed by Mann-Whitney U test. All data shown are median ± interquartile range. Lens: *P*=0.7, WT vs *Pank4*$^{-/-}$, n=3; Retina: *P*=0.1, WT vs *Pank4*$^{-/-}$, n=3; Brain: *P*=0.4, WT vs *Pank4*$^{-/-}$, n=3. (**B**) The protein level and quantification of acetyl CoA carboxylase (ACC) in WT and *Pank4*$^{-/-}$ lens. Data were analyzed by Mann-Whitney U test. All data shown are median ± interquartile range. *P*=0.0286, WT vs *Pank4*$^{-/-}$, n=4.

© 2022. Li X et al. Published online at http://www.aginganddisease.org/EN/10.14336/AD.2023.0216-1

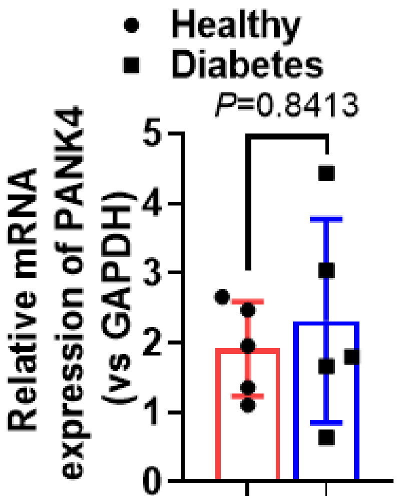

Figure 3. Relative mRNA of Pank4 in healthy and diabetes patients. Data were analyzed by Mann-Whitney U test. All data shown are median ± interquartile range. *P*=0.8413, WT vs *Pank4*$^{-/-}$, n=5.

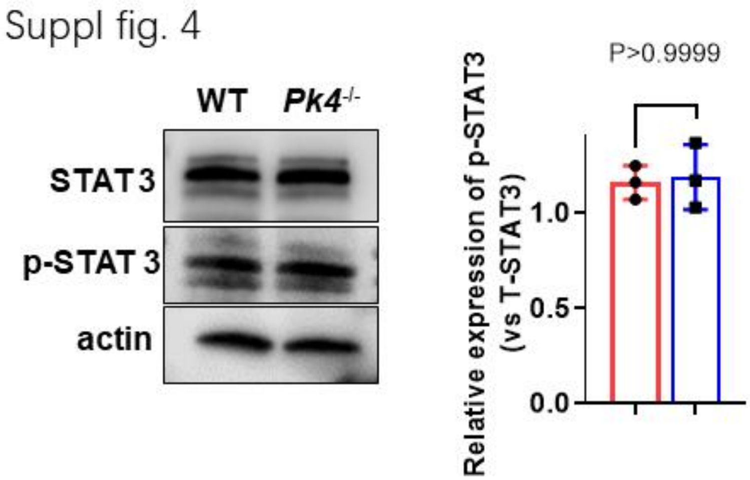

Figure 4. Protein levels of total-Stat3 and phosphor-stat3 in WT and *Pank4*$^{-/-}$ mice lens epithelium. Data were analyzed by Mann-Whitney U test. All data shown are median±interquartile range. *P*>0.9999, WT vs *Pank4*$^{-/-}$, n=3.

© 2022. Li X et al. Published online at http://www.aginganddisease.org/EN/10.14336/AD.2023.0216-1

SUPPLEMENTARY DATA

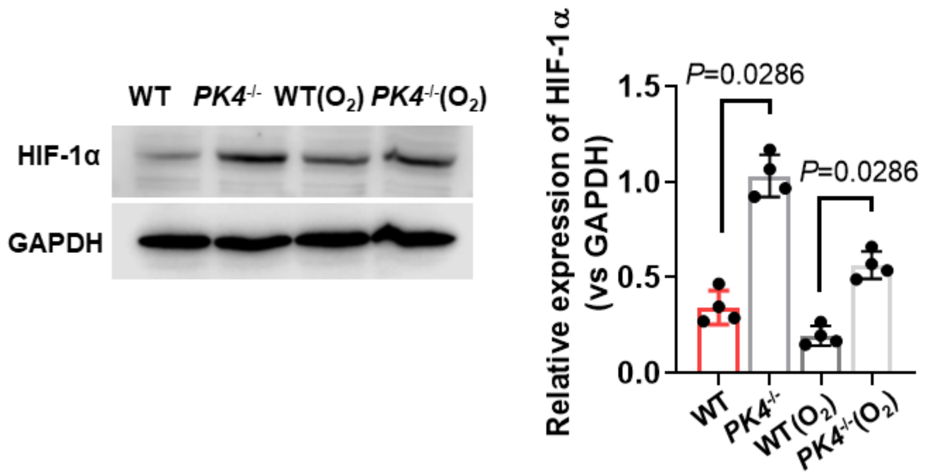

Figure 5. Protein levels of PKM2 and HIF-1α in WT and *Pank4*$^{-/-}$ mice lens with and without 70% oxygen treatment for continuous 7 days. Data were analyzed by Mann-Whitney U test. All data shown are median ± interquartile range. *P*=0.0286, WT *vs. Pank4*$^{-/-}$, n=4; *P*=0.0286, WT(O2) *vs. Pank4*$^{-/-}$(O2), n=4.

Supplementary Table 1. Antibody list.

Antibody	Brand	Cat No.	Host	Reactivity	Dilution
PANK4 (D8N2c)	CST	12055	Rabbit	Human, Mouse, Rat, Monkey	WB=1：1000
PANK4	abnova	ABN-HOOO55229	Rabbit	Human, mouse, Rat	WB=1：1000
PANK4	Bioss	BS-8340R	Rabbit	Human, Mouse, Rat	WB=1:1000
PKM2 (D78A4)	cst	4053	Rabbit	Human, Mouse, Rat, Monkey	WB=1:1000; IF=1:100
PKM2	Bioss	bs-0102M	Mouse	Human, Mouse, Rat, Pig, Cow, Horse, Rabbit	IF=1:100-500
n-cadherin(13A9)	CST	14215S	Mouse	Human, Mouse, Rat, Monkey	WB=1:1000; IF=1:500
college-1	Millipore	3243375	Rabbit	Mouse	WB=1:1000;
a-sma	CST	48938	Mouse	Human, Mouse, Rat	WB=1:1000;
p PKM2 (phospho Y105)	abcam	ab156856	Rabbit	Mouse	WB=1ug/ml
p PKM2 (ser37)	invitrogen	PA5-37684	Rabbit	Human, Mouse	WB=1:1000;
HIF-1α (D1S7W)	CST	36169	Rabbit	Human, Mouse, Monkey	WB=1:1000;
HIF-2α (D6T8V)	CST	59973	Rabbit	Human	WB=1:1000;
HIF-1β/ARNT (D28F3)	CST	5537	Rabbit	Human, Mouse, Rat, Monkey	WB=1:1000;
PFKP (D4B2)	CST	8164	Rabbit	Human, Monkey	WB=1:1000;
LDHA(C4B5)	CST	3582	Rabbit	Human, Monkey	WB=1:1000;
PDHK1 (C47H1)	CST	3820	Rabbit	Human, Mouse, Rat, Monkey	WB=1:1000;
PFKB3(D7H4Q)	CST	13123	Rabbit	Human, Mouse, Rat, Monkey	WB=1:1000;

© 2022. Li X et al. Published online at http://www.aginganddisease.org/EN/10.14336/AD.2023.0216-1

SUPPLEMENTARY DATA

Pyruvate Dehydrogenase (C54G1)	CST	3205	Rabbit	Human, Mouse, Rat, Monkey	WB=1:1000;
PKM1 (D30G6)	CST	7067	Rabbit	Human, Mouse	WB=1:1000;
GOAT ANTI-Rabbit igG/HRP antibody	Bioss	bs-0295G-HRP		Rabbit	WB=1:10000;
GOAT ANTI-Mouse igG/HRP antibody	Bioss	bs-0296G-HRP		Mouse	WB=1:10000;
TGF-β2	GenScript	Z03429	Human		
Stat3(D3Z2G)	CST	12640	Rabbit	Human, Mouse, Rat, Monkey	WB=1:1000;
Phospho-Stat3 (Tyr705) (D3A7)	CST		Rabbit	Human, Mouse, Rat, Monkey	WB=1:1000;
Alexa Fluor® 594-AffiniPure Goat Anti-Rabbit IgG (H+L)	Jackson	111-585-003		Rabbit	IF=1:500
Alexa Fluor® 488-AffiniPure Goat Anti-Rabbit IgG (H+L)	Jackson	111-545-003		Rabbit	IF=1:500
Alexa Fluor® 647-AffiniPure Goat Anti-Rabbit IgG (H+L)	Jackson	111-605-003		Rabbit	IF=1:500
Alexa Fluor® 594-AffiniPure sheep Anti-mouse IgG (H+L)	Jackson	515-585-003		Mouse	IF=1:500

© 2022. Li X et al. Published online at http://www.aginganddisease.org/EN/10.14336/AD.2023.0216-1
